# Comparative evaluation of amniotic membrane and titanium-prepared platelet-rich fibrin in root coverage: A randomized split-mouth clinical trial

**DOI:** 10.1016/j.jobcr.2025.07.002

**Published:** 2025-07-10

**Authors:** Devadharshini Chandrasekar, Burnice Nalina Kumari Chellathurai, Jaideep Mahendra, Vijayalakshmi Rajaram

**Affiliations:** Department of Periodontics, Meenakshi Ammal Dental College and Hospital, Meenakshi Academy of Higher Education and Research , Chennai, Tamil Nadu, India

## Abstract

**Background:**

Gingival recession, particularly Cairo's RT1 and RT2 defects, presents a persistent challenge in periodontal therapy. The comparative effectiveness of soft tissue regenerative materials specifically Amniotic Membrane and Titanium-Prepared Platelet-Rich Fibrin (T-PRF) in the microsurgical management of these defects using the coronally advanced flap (CAF) technique remains inadequately explored.

**Aim:**

To clinically evaluate and compare the efficacy of Amniotic Membrane and T-PRF in the treatment of isolated Cairo's RT1 and RT2 gingival recession defects using the CAF technique.

**Materials and methods:**

A randomized, split-mouth clinical trial was conducted involving 20 patients with bilateral Cairo's RT1 and RT2 recession defects. A total of 46 sites were treated—23 with Amniotic Membrane (Group A) and 23 with T-PRF (Group B), using the CAF technique under surgical magnification. Clinical parameters assessed at baseline, 90 days, and 180 days included recession depth and width, probing pocket depth, clinical attachment level, width of keratinized gingiva, gingival thickness, and mean root coverage.

**Results:**

Both treatment modalities resulted in significant clinical improvements over time. However, Group B (T-PRF) demonstrated statistically superior outcomes in recession depth reduction, clinical attachment level gain, increased keratinized tissue width, greater gingival thickness, and higher mean root coverage compared to Group A (Amniotic Membrane).

**Conclusion:**

T-PRF exhibited enhanced regenerative potential over Amniotic Membrane in the management of isolated Cairo's RT1 and RT2 recession defects when used in conjunction with the CAF technique. These findings support the clinical utility of T-PRF as an effective soft tissue grafting material in periodontal plastic surgery.

## 1 Introduction

Gingival recession (GR) is a condition characterized by the root surface becoming exposed to the oral environment, as the gingival margin recedes apically from its normal position at the cementoenamel junction.^1^ It can be localized or generalized, leading to issues like dentine hypersensitivity, root caries, non-carious cervical lesions (NCCLs), and aesthetic concerns. Effective management involves identifying and addressing underlying causes. Persistent GR may require periodontal aesthetic treatments, like root coverage procedures, aiming to cover exposed roots, reduce hypersensitivity, and meet aesthetic preferences.

Classification systems play a crucial role in facilitating the systematic study of disease etiology, pathogenesis, and treatment approaches. Cairo et al. introduced a treatment-oriented classification system which represents a treatment-oriented approach that centres on the assessment of attachment loss along the buccal and interproximal surfaces, providing clinicians with valuable guidance for periodontal treatment planning. Cairo's Classification categorizes gingival recession based on its severity and extent, with RT 1 and RT 2 referring to recession defects that do not extend to the mucogingival junction.^2^

The coronally advanced flap (CAF) is a standout surgical technique for treating localized gingival recessions, known for its effectiveness in improving both functional and aesthetic outcomes.^3^ Introduced by Shanelec and Tibbets in 1996, microsurgical principles emphasize precise techniques for optimal aesthetic and functional restoration.^4^ Utilizing specialized instruments and magnification, microsurgery enables meticulous tissue manipulation and suturing at a microscopic level, making CAF combined with magnification a cornerstone in modern periodontal therapy. To enhance the success of microsurgical CAF procedures, adjunctive regenerative materials are often used to promote soft tissue regeneration and optimize outcomes. While the connective tissue graft (CTG) is the gold standard due to its histological resemblance to native gingival tissue and clinical efficacy, researchers explore alternatives to address challenges like donor site morbidity and availability.^5^

Platelet-rich fibrin (PRF), pioneered by Choukroun et al. in 2001, enhances periodontal root coverage procedures by promoting tissue repair through cell migration, proliferation, and growth factor release. As PRF technology evolves, its applications in root coverage procedures improve, reflecting a quest for better outcomes. Concerns about silica activation in PRF prepared with glass tubes led to the development of titanium-prepared PRF (T-PRF) by Tunalı et al. in 2014.^6^ T-PRF, a third-generation platelet concentrate, eliminates silica-related adverse effects, displaying a more compact fibrin network and an extended resorption period, enhancing periodontal outcomes.^7^

Amniotic membrane (AM) offers an alternative to platelet concentrates in medical procedures, addressing issues like blood abstinence and time-consuming preparations. Derived from the placenta, AM contains pluripotent cells, growth factors, and proteins, making it valuable across medical fields.^8^ Gurinsky introduced AM in gingival root coverage procedures, harnessing its rich content of fibronectin, laminin, collagen, and cytokines.^9^ Amniotic Membrane promotes cellular migration and proliferation, enhances wound healing, reduces inflammation, minimizes scarring, exhibits antibacterial properties, alleviates pain, and serves as a natural biological barrier.^10^

Though past studies have reported significant outcomes using T-PRF and Amniotic membrane individually in perioplastic procedures, the efficacy of both the membranes have never been compared so far. By elucidating the strengths and limitations of these soft tissue regenerative materials in the microsurgical management of denuded root surface, the present study aims to comparatively evaluate the effectiveness of Amniotic membrane versus T-PRF membrane as a soft tissue regenerative material using coronally advanced technique in the treatment of bilateral Cairo's RT1 & RT2 isolated gingival recession defects in the presence of magnification.

## 2 MATERIALS and methods

### 2.1 Ethical approval and patient selection

**Fig. 1:**
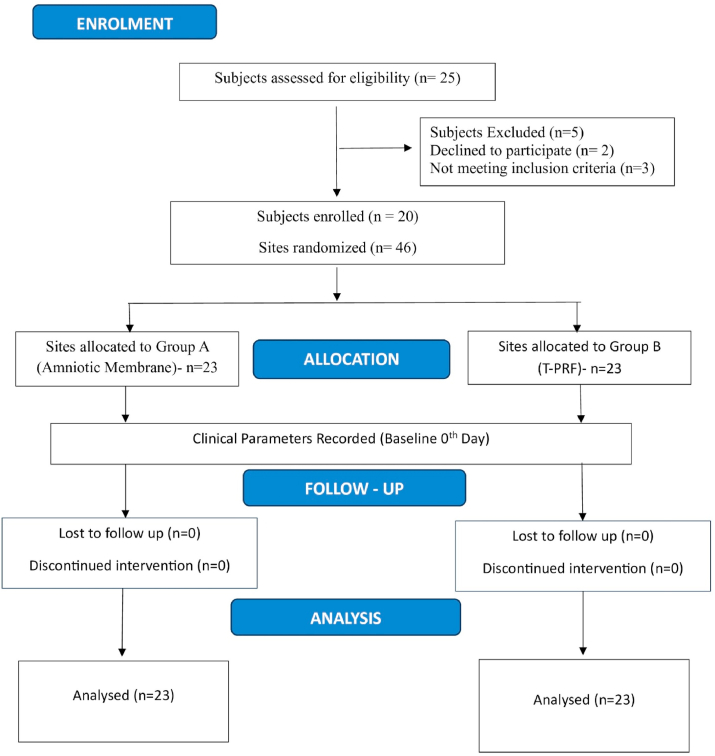
Study design Flowchart.

This randomized split-mouth clinical study was conducted following the approval of the Institutional Ethical Committee (IEC No. MADC/IEC-II/024/2023). The study adhered to the principles of the Declaration of Helsinki, and was registered with the Clinical Trials Registry India (CTRI) under the number [CTRI/2025/04/084986]. Patients were selected from the outpatient department of Periodontology, Meenakshi Ammal Dental College, Meenakshi Academy of Higher Education & Research, Chennai. A total of 20 systemically healthy individuals, including both males and females, presenting with bilateral isolated gingival recession defects classified as Cairo's RT1 and RT2 in the maxillary or mandibular anterior and premolar region were recruited. Patients were randomly allocated into two groups using the coin toss method. Written informed consent was obtained from all participants prior to study enrolment (Fig. 1).

### 2.2 Inclusion and exclusion criteria

The inclusion criteria comprised patients above 18 years of age with bilateral RT1 and RT2 isolated gingival recession defects, recession depth ≥2 mm, gingival thickness >1 mm, width of keratinized gingiva ≥2 mm, probing depth ≤3 mm, and good oral hygiene maintenance. Patients were excluded if they presented with recession defects associated with demineralization, caries, deep abrasion, restorations, or pulpal pathology, evidence of radiographic bone loss or periodontal disease, a history of periodontal surgery in the defect area within the past year, or if they were on medications that could interfere with healing. Pregnant or lactating women, alcoholics, smokers, and medically compromised individuals were also excluded.

### 2.3 Standardization and examiner calibration

Standardization of clinical measurements was achieved using an acrylic stent, ensuring consistent positioning and accurate measurement of clinical parameters. Two examiners, Dr. Devadharshini Chandrasekar (D.C.) and Dr. Burnice Nalina Kumari Chellathurai (BNK.C), were responsible for performing all clinical assessments. Both examiners underwent calibration to minimize inter-examiner variability, ensuring the reliability and consistency of measurements. To eliminate bias, a blinding method was employed throughout the study: the examiners were unaware of the treatment groups assigned to each patient, and the statistician analyzing the data was also blinded to group allocations. This rigorous blinding procedure helped maintain objectivity and impartiality in both the clinical evaluation and data analysis.

### 2.4 Clinical parameters and assessment

The clinical parameters assessed in this study included Oral Hygiene Index-Simplified (OHI-S), Gingival Index (GI), recession depth, recession width, probing pocket depth (PPD), clinical attachment level (CAL), keratinized gingiva width (KGW), gingival thickness (GT), mean root coverage (MRC), and wound healing index (WHI). These parameters were evaluated and recorded at baseline (day 0), 30 days, 90 days, and 180 days postoperatively.

### 2.5 Study design and treatment groups

**Fig. 2:**
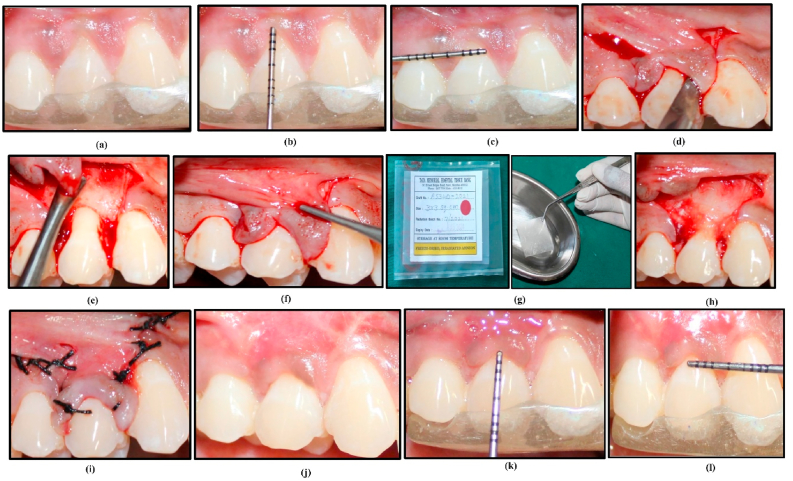
Clinical sequence of the coronally advanced flap (CAF) procedure with Amniotic Membrane for root coverage in Group A. 2a: "Preoperative photograph showing isolated gingival recession involving tooth 14." 2b: "Measurement of recession height prior to surgery." 2c: "Measurement of recession width prior to surgery." 2d: "Sulcular and vertical releasing incisions made to define the surgical area." 2e: "Flap elevated to facilitate coronal advancement." 2f: "Flap passively positioned in a coronally advanced manner." 2g: "Preparation of the Amniotic Membrane prior to placement." 2h: "Amniotic Membrane positioned within the elevated flap." 2i: "Suturing of the coronally advanced flap to stabilize the membrane." 2j: "Postoperative photograph at 180 days showing root coverage of tooth 14." 2k: "Measurement of recession height at 180-day follow-up." 2l: "Measurement of recession width at 180-day follow-up."

This study followed a split-mouth design, wherein 20 patients with bilateral RT1 and RT2 isolated gingival recession defects were randomly assigned to two treatment groups. In Group A, 23 sites were treated with an amniotic membrane in conjunction with a coronally advanced flap (CAF) technique under magnification (Figure-2). In Group B, 23 sites were treated with a titanium-prepared platelet-rich fibrin (T-PRF) membrane using the CAF technique under magnification (Figure - 3).

**Fig. 3:**
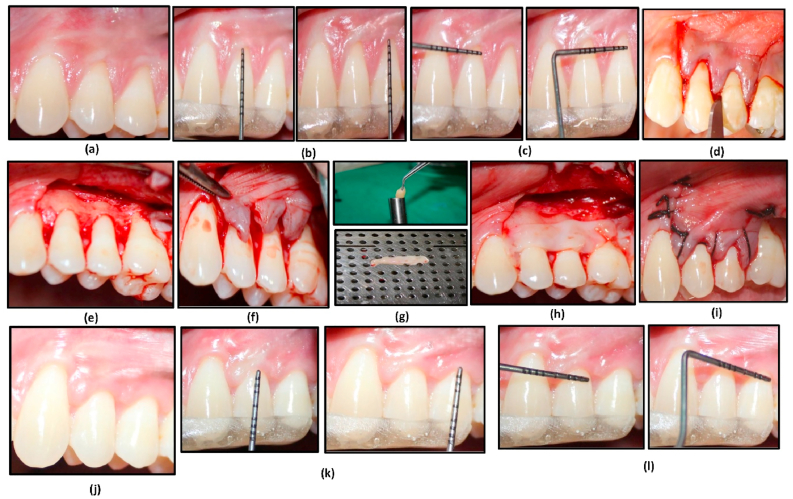
Clinical sequence of the coronally advanced flap (CAF) procedure with Titanium-Prepared Platelet-Rich Fibrin (T-PRF) for root coverage in Group B. 3a: "Preoperative photograph showing gingival recession involving teeth 24 and 25." 3b: "Measurement of recession height prior to surgery." 3c: "Measurement of recession width prior to surgery." 3d: "Sulcular and vertical releasing incisions made to define the surgical area." 3e: "Flap elevated to allow coronal advancement." 3f: "Flap passively positioned in a coronally advanced manner." 3g: "Preparation of Titanium-Prepared Platelet-Rich Fibrin (T-PRF) prior to placement." 3h: "T-PRF placed within the elevated flap." 3i: "Suturing of the coronally advanced flap to secure the T-PRF." 3j: "Postoperative photograph at 180 days showing root coverage of teeth 24 and 25." 3k: "Measurement of recession height at 180-day follow-up." 3l: "Measurement of recession width at 180-day follow-up."

### 2.6 Phase I therapy and pre-surgical procedures

All patients underwent thorough scaling and root planing, and baseline clinical parameters were recorded on the same day. Amniotic membrane allografts used in this study were procured from the Tissue Bank, Tata Memorial Hospital, Mumbai, India. T-PRF was prepared following the protocol described by Tunali et al.^6^ Briefly, 20 mL of blood was drawn from the antecubital vein and transferred into titanium-coated sterile test tubes without anticoagulant. The blood was centrifuged at 2800 rpm for 12 min. The T-PRF clot was then retrieved from the middle portion of the tube, separated from the red blood cell layer without the use of scissors, and compressed in sterile gauze to form a membrane of uniform thickness.

### 2.7 Surgical procedure

All surgeries were performed by the same examiner. Patients were instructed to perform a pre-procedural rinse with 0.12 % chlorhexidine gluconate for one to two min. Local anesthesia was administered using 2 % lignocaine hydrochloride with 1:80,000 adrenaline. Two horizontal incisions were made mesially and distally from the CEJ up to 1 mm beyond the proximal line angles of the adjacent teeth, maintaining the integrity of the interdental papilla. Vertical releasing incisions were placed interdentally on the labial aspect, extending beyond the mucogingival junction. A sulcular incision was made, and a trapezoidal mucoperiosteal flap was raised via blunt dissection. Partial-thickness dissection was performed apically to the recession defect, and the buccal portion of the intact papilla was de-epithelialized to serve as a recipient site for the graft material in the coronally repositioned flap.

In Group A, the amniotic membrane was placed over the prepared site, whereas in Group B, the T-PRF membrane was positioned accordingly. The buccal flap was coronally advanced and secured with 6-0 Mersilk sutures using a horizontal sling technique, ensuring proper flap adaptation over the enamel surface. Interrupted sutures were placed to close the vertical releasing incisions, and a non-eugenol periodontal dressing (Coe-Pack) was applied. Patients were instructed to avoid rinsing or spitting for 24 h, refrain from brushing in the surgical area for three days, adhere to a soft diet, and take prescribed medications. Postoperative analgesia was provided in the form of Aceclofenac 100 mg with Paracetamol 325 mg, administered twice daily for three days. All surgical procedures were performed under 3.5 × magnification.

### 2.8 Post-surgical follow-up and maintenance

Sutures were removed after 14 days, and all patients were placed on a strict maintenance schedule with follow-up visits at 30, 90, and 180 days postoperatively. At each visit, oral hygiene was reinforced, and all clinical parameters were recorded. The mean root coverage percentage was assessed at 180 days, evaluating the reduction in recession depth and width.

## 3 Statistical analysis

Data was analyzed using SPSS software version 26.0 (IBM Corp., Armonk, NY, USA). Continuous variables were presented as mean ± standard deviation (M ± SD) and categorical variables as frequencies (n, %). Normality was assessed using the Shapiro-Wilk test, and parametric tests were applied. Intergroup comparisons of OHI-S scores, Gingival Index scores, recession depth, recession width, PPD, CAL, KGW, and GT between the amniotic membrane and T-PRF groups at baseline, 90 days, and 180 days were performed using an independent sample *t*-test. Intragroup comparisons across time points were analyzed using repeated measures ANOVA. Categorical variables, including WHI scores, were compared using the Chi-square test. A p-value <0.05 was considered statistically significant.

## 4 Results

The Oral Hygiene Index-Simplified (OHI-S) scores showed a progressive and statistically significant reduction over time in both groups (p < 0.001). However, since the study followed a split-mouth design, no significant differences were observed between the groups at any time point. Similarly, Gingival Index (GI) scores demonstrated a mild reduction from baseline (0th day) to the 90th day, with no further decrease at the 180th day. This reduction was not statistically significant (Table. 1).

**Table 1:** Comparison of oral hygiene index–Simplified (OHI-S) and gingival index scores in participants operated with T-PRF (group B) and amniotic membrane (group A) at baseline, 90th day and 180th day.

Study groups	OHI-S scores			p-value^b^
	Baseline	90th Day	180th Day	
	M ± S.D	M ± S.D	M ± S.D	
**T-PRF (Group B)**	0.86 ± 0.34	0.77 ± 0.31	0.72 ± 0.29	**<0.001**^c^
**Amniotic Membrane (Group A)**	0.86 ± 0.34	0.77 ± 0.31	0.726 ± 0.28	**<0.001**^c^
**p-value**^a^	1.0	1.0	1.0	

**Study groups**	**Gingival index scores**			**p-value**^b^
	**Baseline**	**90**th **Day**	**180**th **Day**	
	**M ± S.D**	**M ± S.D**	**M ± S.D**	
**T-PRF (Group B)**	1.09 ± 0.28	1.0 ± 0.0	1.0 ± 0.0	0.16
**Amniotic Membrane (Group A)**	1.09 ± 0.28	1.0 ± 0.0	1.0 ± 0.0	0.16
**p-value**^a^	1.0	1.0	1.0	

a Independent sample *t*-test.

b Repeated Measures ANOVA.

c p-value<0.05 – statistically significant.

Recession depth showed a significant reduction in both groups from baseline (0th day) to the 90th and 180th days (p < 0.001). Sites treated with T-PRF exhibited a greater reduction (2.91 ± 0.66 mm to 0.70 ± 0.10 mm) compared to those treated with the amniotic membrane (2.39 ± 0.49 mm to 0.61 ± 0.18 mm). Intergroup comparisons revealed statistically significant differences at baseline (0th day) (p = 0.01) and the 90th day (p = 0.02), although the difference at the 180th day was not statistically significant. Recession width also showed a significant reduction over time in both groups (p < 0.001). In the T-PRF group, recession width reduced from 2.30 ± 0.55 mm at baseline (0th day) to 0.65 ± 0.11 mm at the 180th day, whereas in the amniotic membrane group, it reduced from 1.83 ± 0.38 mm to 0.57 ± 0.10 mm. A significant difference was noted between the groups at baseline (0th day) (p = 0.02), but not at subsequent time points. The probing pocket depth (PPD) remained constant in both groups throughout the study period (p = 1.00). Clinical attachment level (CAL) improved significantly in both groups over time (p < 0.001). The T-PRF group exhibited a greater reduction from 5.91 ± 0.66 mm to 3.70 ± 0.70 mm at the 180th day, compared to the amniotic membrane group (5.39 ± 0.50 mm to 3.61 ± 0.58 mm). A significant difference was observed between the groups at baseline (0th day) (p = 0.01), but not at later time points. The width of keratinized gingiva (KGW) significantly increased in the T-PRF group (p = 0.04) from 1.91 ± 0.41 mm at baseline (0th day) to 2.13 ± 0.45 mm at the 180th day. The amniotic membrane group showed a minor increase from 1.22 ± 0.42 mm to 1.35 ± 0.48 mm, which was not statistically significant. Intergroup comparisons at all time points were statistically significant (p < 0.001), indicating superior outcomes in the T-PRF group. A significant improvement in gingival thickness (GT) was observed in the T-PRF group (p < 0.001), increasing from 1.35 ± 0.48 mm at baseline (0th day) to 2.00 ± 0.00 mm at the 180th day. The amniotic membrane group exhibited a non-significant increase from 1.09 ± 0.29 mm to 1.26 ± 0.44 mm. Intergroup comparisons were statistically significant at all time points (p < 0.001), favoring the T-PRF group. Intragroup comparisons of all outcome parameters demonstrated statistically significant improvements within both treatment groups over time (Table. 2).

**Table 2:** Comparisons of Clinical parameters between T-PRF (Group B) and Amniotic Membrane (Group A) at Baseline, 90th Day and 180th Day.

Outcome parameter	Time frame	T-PRF (Group B)		Amniotic membrane (Group A)	
		Mean difference	p-value	Mean difference	p-value
**Recession depth**	Baseline vs 90th day	1.74	**<0.001∗**	1.13	**<0.001∗**
	Baseline vs 180th day	2.2	**<0.001∗**	1.78	**<0.001∗**
	90th day vs 180th day	0.47	**<0.001∗**	0.65	**<0.001∗**
**Recession width**	Baseline vs 90th day	1.52	**<0.001∗**	1.0	**<0.001∗**
	Baseline vs 180th day	1.65	**<0.001∗**	1.26	**<0.001∗**
	90th day vs 180th day	0.13	0.26	0.26	**0.03∗**
**Clinical attachment level**	Baseline vs 90th day	1.73	**<0.001∗**	1.13	**<0.001∗**
	Baseline vs 180th day	2.21	**<0.001∗**	1.78	**<0.001∗**
	90th day vs 180th day	0.48	**<0.001∗**	0.65	**<0.001∗**
**Keratinized gingival width**	Baseline vs 90th day	−0.17	**0.04∗**	0.0	1.0
	Baseline vs 180th day	−0.21	**0.04∗**	−0.13	0.08
	90th day vs 180th day	−0.04	0.57	−0.13	0.08
**Gingival thickness**	Baseline vs 90th day	−0.39	**0.001∗**	−0.17	**0.01∗**
	Baseline vs 180th day	−0.65	**0.001∗**	−0.17	**0.01∗**
	90th day vs 180th day	−0.26	**0.01∗**	0.0	1.0

-Paired *t*-test; ∗p-value<0.05 – statistically significant.

The mean percentage of root coverage (MRC) was significantly higher in the T-PRF group at both the 90th day (64.85 ± 27.05 %) and the 180th day (78.25 ± 22.71 %) compared to the amniotic membrane group (47.09 ± 31.57 % at the 90th day and 70.46 ± 23.80 % at the 180th day), with statistically significant intergroup differences at both time points (p = 0.04 and p = 0.03, respectively) (Figure - 4). The wound healing index (WHI) scores were comparable between the groups, with 91.3 % of patients in the T-PRF group achieving optimal healing (score 1) compared to 78.2 % in the amniotic membrane group. However, this difference was not statistically significant (p = 0.20) (Table-3).

**Fig. 4:**
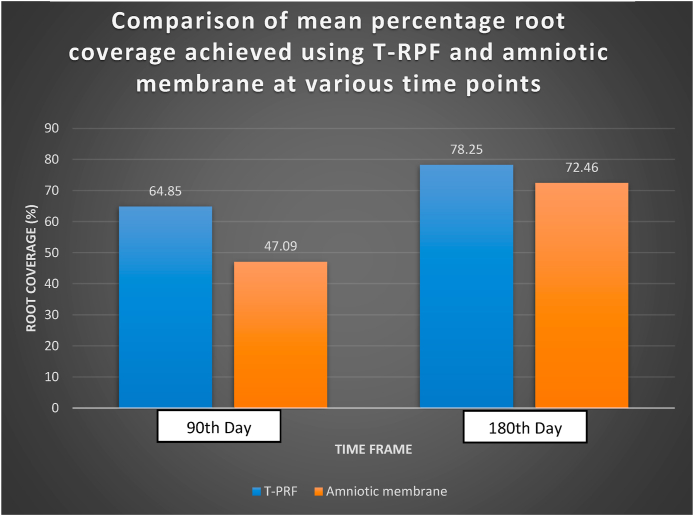
Comparison of mean percentage root coverage achieved using T-RPF (Group B) and Amniotic membrane (Group A) at various time points.

**Table 3:** Comparison of mean root coverage (%) and wound healing index scores between T-PRF (group B) and amniotic membrane (group A) at baseline, 90th day and 180th day.

Study groups	Mean root coverage (%)			p-value^b^
	Baseline	3 months	6 months	
	M ± S.D	M ± S.D	M ± S.D	
**T-PRF**	–	64.85 ± 27.05	78.25 ± 22.71	**<0.001**^d^
**Amniotic membrane**	–	47.09 ± 31.57	70.46 ± 23.80	**<0.001**^d^
**p-value**^a^	**-**	**0.04**^d^	**0.03**^d^	
aIndependent sample *t*-test; bPaired *t*-test;				
	**Wound healing index scores**		**Total**	**p-value**^c^
	**Score 1**	**Score 2**		
**T-PRF n (%)**	21 (91.3)	2 (8.7)	23 (100)	0.20 (NS)
**Amniotic membrane n (%**	18 (78.2)	5 (21.8)	23 (100)	

-Chi square test.

a Independent sample *t*-test.

b Paired *t*-test.

c Chi square test.

d p-value<0.05 – statistically significant.

T-PRF consistently produced superior outcomes in terms of recession depth and width reduction, increased clinical attachment gain, improved keratinized gingival width, and greater gingival thickness when compared to the amniotic membrane. These findings suggest that T-PRF offers enhanced regenerative potential in the microsurgical management of isolated gingival recession defects.

## 5 Discussion

Gingival recession refers to the apical displacement of the gingival margin beyond the cementoenamel junction (CEJ), leading to the exposure of the root surface. Cairo et al. framed a classification system that has improved the understanding and treatment planning for recession defects, with RT1 (no interproximal attachment loss) and RT2 (interproximal attachment loss equal to or less than the buccal attachment loss) being the focus of this study.^2^ A split-mouth randomized clinical design was employed to compare the efficacy of Amniotic Membrane (AM) and Titanium-Prepared Platelet-Rich Fibrin (T-PRF) in conjunction with the coronally advanced flap (CAF) technique, minimizing inter-subject variability and enhancing statistical power. CAF remains the gold standard for root coverage, providing optimal wound healing and attachment gain, particularly when combined with biomaterials. Amniotic membrane, derived from the human placenta, is rich in growth factors and extracellular matrix components that enhance epithelialization, angiogenesis, and anti-inflammatory responses, making it a promising adjunct for periodontal regeneration. Recent systematic review and meta-analysis confirmed the efficacy of AM when used in combination with the coronally advanced flap, highlighting improved clinical outcomes in the treatment of gingival recession.^11^ Similarly, T-PRF, a second-generation platelet concentrate, forms a dense fibrin matrix with sustained release of growth factors like Platelet-Derived Growth Factor (PDGF) and Transforming Growth Factor-β1 (TGF-β1), facilitating angiogenesis, fibroblast proliferation, and tissue regeneration.^7^ cost-effectiveness, ease of preparation, and autologous nature make it a valuable adjunct to the coronally advanced flap (CAF) technique in periodontal plastic surgery. This study aimed to evaluate the clinical efficacy of AM and T-PRF as soft tissue regenerative materials in treating bilateral Cairo's RT1 and RT2 gingival recession defects using the CAF technique under magnification.

The split-mouth study demonstrated a statistically significant reduction in OHI-S scores from baseline to the 90th and 180th days (p-value <0.001) in both T-PRF and amniotic membrane groups. Baseline scores were identical (0.86 ± 0.34), with reductions at 90 days (0.77 ± 0.31) and 180 days (0.72 ± 0.29 for T-PRF and 0.726 ± 0.28 for amniotic membrane), highlighting improved oral hygiene maintenance following periodontal intervention. Garzón et al. emphasized the immunomodulatory properties of biomaterials, which contribute to a favourable healing environment and enhanced periodontal health outcomes.^12^ Similarly, Santonocito et al. ireported that scaffolds and biomaterials play a pivotal role in periodontal tissue regeneration, facilitating improved clinical stability.^13^ These findings align with the current study, underscoring the role of T-PRF and amniotic membrane as effective adjuncts in enhancing oral hygiene adherence and promoting long-term periodontal stability.

The comparison of gingival index scores in participants operated with T-PRF and amniotic membrane at baseline, 90th day, and 180th day showed identical values due to the split-mouth design. A mild reduction in scores was noted from baseline (1.09 ± 0.28) to the 90th day (1.0 ± 0.0), with no further changes by the 180th day, and the overall reduction remained statistically non-significant (p = 0.16). This suggests comparable efficacy of T-PRF and amniotic membrane in maintaining gingival health postoperatively. Petrescu et al. emphasized that platelet-rich fibrin (PRF) enhances gingival tissue regeneration by promoting angiogenesis and reducing inflammation, thereby supporting gingival stability.^14^ Similarly, Chaitra et al. reported that both amniotic membrane and PRF demonstrated effective outcomes in the management of gingival recession, with comparable improvements in gingival health.^15^ These findings align with the current study, underscoring the equal potential of T-PRF and amniotic membrane as adjuncts in achieving and maintaining gingival health when incorporated into a comprehensive periodontal treatment protocol.

The comparison of recession depth between T-PRF and amniotic membrane at baseline, 90th day, and 180th day revealed significant reductions in both groups over the study period. Sites treated with T-PRF showed a reduction in mean recession depth from 2.91 ± 0.66 at baseline to 1.26 ± 0.75 at the 90th day and further to 0.70 ± 0.10 at the 180th day (p < 0.001). Similarly, sites treated with amniotic membrane exhibited a reduction from 2.39 ± 0.49 at baseline to 0.78 ± 0.19 at the 90th day and to 0.61 ± 0.18 at the 180th day (p < 0.001). Intergroup comparisons demonstrated significant differences at baseline (p = 0.01) and the 90th day (p = 0.02), but not at the 180th day (p = 0.12). Notably, T-PRF achieved greater overall reduction in recession depth (from 2.91 to 0.70 mm) compared to the amniotic membrane (from 2.39 to 0.61 mm), underscoring its superior performance. Bhattacharya et al. highlighted the additional benefits of T-PRF combined with a coronally advanced flap (CAF), reporting significant improvements in root coverage and gingival thickness due to the enhanced regenerative potential of T-PRF.^16^ Lubaib et al. conducted a meta-analysis demonstrating the effectiveness of amniotic membrane in reducing gingival recession depth, attributing its success to its bioactive properties that promote wound healing and tissue regeneration.^17^ These findings align with the current study, reinforcing that while both T-PRF and amniotic membrane are effective in reducing recession depth, T-PRF may offer an edge in achieving greater tissue regeneration over time.

The comparison of recession width between T-PRF and amniotic membrane at baseline, 90th day, and 180th day revealed significant reductions in both groups over the study period. In the T-PRF group, the mean recession width decreased from 2.30 ± 0.55 at baseline to 0.78 ± 0.19 at the 90th day and further reduced to 0.65 ± 0.11 at the 180th day (p < 0.001). Similarly, in the amniotic membrane group, the mean recession width reduced from 1.83 ± 0.38 at baseline to 0.83 ± 0.38 at the 90th day and further to 0.57 ± 0.10 at the 180th day (p < 0.001). Intergroup comparisons revealed a significant difference at baseline (p = 0.02) but not at the 90th day (p = 0.63) or the 180th day (p = 0.81). Despite the absence of statistically significant differences at later time points, the consistent reduction in recession width observed with T-PRF highlights its clinical predictability and sustained efficacy compared to amniotic membrane. The findings of Uzun et al. support this observation as their study demonstrated the effectiveness of T-PRF in managing multiple gingival recessions with significant improvements in both recession depth and width. They attributed the superior outcomes to the regenerative potential and structural stability provided by T-PRF.^18^ Nath et al. reported significant reductions in gingival recession width with the use of a coronally advanced flap combined with amniotic membrane, emphasizing its bioactive role in promoting wound healing and epithelialization.^19^ These studies corroborate the present findings, suggesting that while both T-PRF and amniotic membrane are effective in managing gingival recession width, T-PRF offers an additional advantage due to its robust regenerative properties and ability to maintain long-term clinical outcomes.

The comparison of probing pocket depth (PPD) between T-PRF and amniotic membrane showed no changes throughout the study period with both groups maintaining a stable mean PPD of 3 ± 0.0 mm at baseline, 90th day, and 180th day (p = 1.00). This stability aligns with findings by Eminoglu et al. reported no significant changes in PPD despite T-PRF's regenerative benefits in periodontal intrabony defects, attributing the results to enhanced soft tissue healing.^20^ Law et al. demonstrated that amniotic membrane promotes periodontal regeneration while maintaining stable PPD, supporting its role in preserving periodontal health.^21^ These studies confirm that both T-PRF and amniotic membrane effectively manage gingival recession without altering probing pocket depth.

The comparison of clinical attachment levels (CAL) between T-PRF and amniotic membrane demonstrated significant reductions in CAL from baseline to the 180th day, indicating effective periodontal regeneration in both groups. In sites treated with T-PRF, the CAL decreased from 5.91 ± 0.66 mm at baseline to 4.17 ± 0.93 mm at 90th day, and further to 3.70 ± 0.70 mm at 180th day (p < 0.001). Similarly, in sites treated with amniotic membrane, CAL reduced from 5.39 ± 0.50 mm at baseline to 4.26 ± 0.75 mm at 90th day, and further to 3.61 ± 0.58 mm at 180th day (p < 0.001). Intergroup comparisons revealed a statistically significant difference at baseline (p = 0.01), but no significant differences were observed at the 90th and 180th days. These findings suggest that both T-PRF and amniotic membrane effectively promoted clinical attachment gains, but T-PRF demonstrated slightly superior outcomes in CAL improvement over time. The findings align with a study done by Gummaluri et al. in which they concluded superior clinical attachment gains with T-PRF compared to leucocyte platelet-rich fibrin in intra-bony defects.^22^ Singi & Shah in their randomised controlled trial confirmed the efficacy of amniotic membrane in enhancing clinical attachment levels.^23^ Thus, while both materials show promise, T-PRF appears to offer more predictable and superior clinical outcomes in terms of clinical attachment level gains.

The comparison of keratinized gingival width achieved between T-PRF and amniotic membrane demonstrated significant differences at baseline, 90th day, and 180th day. In sites treated with T-PRF, the keratinized gingival width increased from 1.91 ± 0.41 mm at baseline to 2.09 ± 0.29 mm at the 90th day, and further increased to 2.13 ± 0.45 mm at the 180th day (p < 0.001). In contrast, sites treated with amniotic membrane exhibited a more modest increase from 1.22 ± 0.42 mm at baseline to 1.35 ± 0.48 mm at the 180th day, with the improvement being statistically non-significant (p = 0.08). Intergroup comparisons revealed a statistically significant difference between T-PRF and amniotic membrane at all time points (p < 0.001). These findings align with Vaswani and Bajaj in their study reported the effectiveness of T-PRF in increasing keratinized gingival width, emphasizing its role in soft tissue regeneration.^24^ Chakraborthy et al. demonstrated the efficacy of amnion and chorion allografts in combination with coronally advanced flap techniques for enhancing keratinized gingival width.^25^ However, the current study reinforces T-PRF's greater ability to significantly enhance keratinized gingival width over time, underscoring its superior efficacy in periodontal soft tissue regeneration.

The comparison of gingival thickness between T-PRF and amniotic membrane demonstrated significant differences in tissue regeneration over the study period. In sites treated with T-PRF, the mean gingival thickness increased from 1.35 ± 0.48 mm at baseline to 1.74 ± 0.45 mm at 90th day, and further increased to 2.0 ± 0.0 mm at 180th day (p < 0.001). In contrast, the amniotic membrane group showed a smaller, statistically non-significant increase in gingival thickness from 1.09 ± 0.29 mm at baseline to 1.26 ± 0.44 mm at 180th day (p = 0.13). Intergroup comparisons revealed statistically significant differences in gingival thickness between T-PRF and amniotic membrane at all time points (p < 0.001). These findings align with the study by Ozkan Şen and Oncu which demonstrated T-PRF's superior ability to increase gingival thickness in multiple maxillary gingival recessions compared to subepithelial connective tissue grafts.^3^ Bolla et al. observed that amniotic membrane showed limited effectiveness in increasing gingival thickness when used in combination with coronally advanced flap procedures.^26^ Thus, T-PRF is shown to be a more effective material for enhancing gingival thickness, contributing to improved periodontal tissue outcomes.

The comparison of mean root coverage between T-PRF and amniotic membrane consistently demonstrated superior outcomes with T-PRF. At the 90th day, T-PRF achieved a mean percentage root coverage of 64.85 ± 27.05 %, while amniotic membrane achieved 47.09 ± 31.57 %, with a statistically significant difference (p-value = 0.04). By the 180th day, T-PRF continued to show increased root coverage, reaching a mean percentage of 78.25 ± 22.71 %, compared to 70.46 ± 23.80 % for amniotic membrane, maintaining a significant statistical difference (p-value = 0.04). These results align with findings from previous studies. Bhattacharya et al. demonstrated that T-PRF when combined with coronally advanced flap (CAF) procedures, yields superior root coverage outcomes compared to other treatment options.^17^ Lafzi et al. observed that while amniotic membrane demonstrated some improvement, its efficacy was less consistent compared to subepithelial connective tissue grafts in Miller's class I and II gingival recessions.^27^ These findings collectively underscore T-PRF's superior capability for facilitating effective root coverage, making it a more favourable option for achieving optimal periodontal soft tissue regeneration.

Post-operative wound healing scores were assessed in patients treated with T-PRF and amniotic membrane. In the T-PRF group, 21 (91.3 %) patients achieved a wound healing score of 1, while 2 (8.7 %) patients had a score of 2. In the amniotic membrane group, 18 (78.2 %) patients had a wound healing score of 1, and 5 (21.8 %) patients had a score of 2. Although a higher percentage of patients in the T-PRF group achieved optimal wound healing scores (score 1), the difference in post-operative wound healing scores between the two groups was not statistically significant (p = 0.20). Ustaoglu et al. demonstrated that both T-PRF and L-PRF similarly enhanced wound epithelization and reduced postoperative discomfort at extraction sockets, with T-PRF showing superior regenerative properties compared to L-PRF and the control group.^28^ Gajul et al. emphasized the advantages of dehydrated human amniotic/chorionic membrane (dHACM) noting its anti-inflammatory properties, angiogenetic potential and ability to promote better healing outcomes in comparison to sites treated without dHAM.^29^ These studies further support the comparable efficacy of T-PRF and amniotic membrane in promoting wound healing.

The subjective observations and collected data over the study timeline have demonstrated the efficacy of both Amniotic Membrane (AM) and Titanium-prepared Platelet-Rich Fibrin (T-PRF) when combined with the Coronally Advanced Flap (CAF) technique under magnification for the treatment of gingival recession. The findings indicate significant improvements in recession depth, recession width, keratinized tissue width, gingival thickness, and clinical attachment level in both treatment groups. The use of the Coronally Advanced Flap technique under magnification, facilitated by loupes, minimized tissue trauma and scarring, contributing to a less invasive surgical procedure. To the best of our knowledge, this is the first randomized controlled trial comparing the effectiveness of AM and T-PRF using the Coronally Advanced Flap technique in treating gingival recession under magnification.

In this study, the mean change observed from baseline to the 180th day revealed that T-PRF consistently outperformed AM in terms of all clinical parameters. T-PRF demonstrated superior results in recession depth, recession width, keratinized tissue width, gingival thickness and clinical attachment level gain. However, it is essential to acknowledge some limitations of the study. The sample size was relatively small, which may impact the generalizability of the findings to a broader population. Additionally, the follow-up period was restricted to six months, limiting the assessment of long-term outcomes. While T-PRF demonstrated promising results, it is associated with higher costs and the requirement for specialized equipment, potentially limiting its accessibility. Conversely, Amniotic Membrane may face challenges due to variability in donor tissue quality and potential immune responses. These limitations could affect the reliability of long-term efficacy comparisons between T-PRF and AM. Further validation through studies with larger sample sizes and extended follow-up durations is necessary to confirm the observed outcomes and establish long-term clinical benefits.

## 6 Conclusion

This study underscores the critical role of biomaterials in periodontal regenerative therapy by comparing the clinical efficacy of Amniotic Membrane (AM) and Titanium-Prepared Platelet-Rich Fibrin (T-PRF) in managing Cairo's RT1 and RT2 gingival recession defects. The findings demonstrated that T-PRF achieved superior outcomes in mean root coverage, gingival thickness, keratinized gingiva width, and recession depth reduction, attributed to its enhanced fibrin architecture and sustained growth factor release. The study highlights the strategic importance of biomaterial selection in periodontal therapy, with T-PRF emerging as the preferred option for optimal functional and aesthetic results, whereas AM remains a viable alternative in specific clinical scenarios. Future large-scale studies with extended follow-up are recommended to further validate these findings and explore the integration of emerging technologies to enhance periodontal regeneration.

## Patient's/guardian's consent

Written informed consent was obtained from all participants prior to their enrollment in the study. Each patient was thoroughly informed about the purpose, procedures, potential risks, benefits, and alternatives involved in the clinical trial, both verbally and through a detailed patient information sheet in their native language. They were given adequate time to ask questions and consider their participation.

All participants were assured of their right to withdraw from the study at any stage without any compromise in the standard of care they would receive. Consent also included approval for the use of anonymized clinical data, photographs, and results for academic and publication purposes. Specific attention was paid to maintaining confidentiality, and no identifying information has been or will be disclosed in any publication or presentation.

## Author contributions

All authors have made substantial contributions to the conception and design of the study. D.C., B.N.K.C., and J.M. were involved in data collection and clinical assessments. D.C., B.N.K.C., and J.M. contributed to the acquisition and interpretation of clinical data. D.C. and B.N.K.C. drafted the manuscript, while all authors critically revised it for intellectual content. D.C. and B.N.K.C. provided oversight and technical guidance throughout the study. V.R. provided guidance on clinical case management and treatment planning. J.M. and B.N.K.C. gave final approval for the manuscript to be published.

## Ethical clearance

This randomized split-mouth clinical trial was conducted in full compliance with ethical standards following approval from the Institutional Ethical Committee (IEC) of Meenakshi Ammal Dental College and Hospital, Chennai, affiliated with the Meenakshi Academy of Higher Education and Research (Deemed to be University), Chennai. The study protocol was approved under IEC reference number **MADC/IEC-II/024/2023**.

All study procedures were conducted in accordance with the ethical principles outlined in the Declaration of Helsinki (2013) and in compliance with the Indian Council of Medical Research (ICMR) ethical guidelines for biomedical research involving human participants. The methodology was designed to uphold patient safety, ensure data confidentiality, and maintain scientific rigor throughout the investigation.

## Sources of funding

This study did not receive any specific grant from funding agencies in the public, commercial, or not-for-profit sectors. The research was entirely self-funded by the investigators as part of academic clinical research conducted within the Department of Periodontics, Meenakshi Ammal Dental College and Hospital, Chennai, Tamilnadu, India.

## Declaration of competing interest

The authors declare that they have no known competing financial interests or personal relationships that could have appeared to influence the work reported in this paper.

## References

[1] Tugnait A., Clerehugh V. (2001). Gingival recession—Its significance and management. J Dent.

[2] Cairo F., Nieri M., Cincinelli S., Mervelt J., Pagliaro U. (2011). The interproximal clinical attachment level to classify gingival recessions and predict root coverage outcomes: an explorative and reliability study. J Clin Periodontol.

[3] Ozkan Sen D., Oncu E. (2023). Splith mouth randomized control trial comparison of T‐PRF and subepithelial connective tissue graft in the treatment of maxillar multiple gingival recessions. J Esthetic Restor Dent.

[4] Da Shanelec, Tibbetts Ls (2000. 1996 Jun). A perspective on the future of periodontal microsurgery. Periodontol.

[5] Aroca S., Molnár B., Windisch P. (2013). Treatment of multiple adjacent miller class I and IIgingival recessions with a modified coronally advanced tunnel (MCAT) technique and a collagen matrix or palatal connective tissue graft: a randomized, controlled clinical trial. J Clin Periodontol.

[6] Tunalı M., Özdemir H., Küçükodacı Z. (2014). A novel platelet concentrate: titanium-prepared platelet-rich fibrin. BioMed Res Int.

[7] Tunalı M., Özdemir H., Küçükodacı Z., Akman S., Fıratlı E. (2013). In vivo evaluation of titanium-prepared platelet-rich fibrin (T-PRF): a new platelet concentrate. Br J Oral Maxillofac Surg.

[8] Lamba S., Dahiya R., Blaggana A. (2022). Comparative evaluation of coronally advanced flap procedure in conjunction with amniotic membrane versus coronally advanced flap with platelet-rich fibrin membrane in patients with miller's class I and II gingival recession defects. Dental Journal of Advance Studies.

[9] Gurinsky B. (2009). A novel dehydrated amnion allograft for use in the treatment of gingival recession: an observational case series. J Implant Adv Clin Dent.

[10] Fetterolf D.E., Snyder R.J. (2012). Scientific and clinical support for the use of dehydrated amniotic membrane in wound management. Wounds.

[11] Abdel-Fatah R., Saleh W. (2024). Efficacy of amniotic membrane with coronally advanced flap in the treatment of gingival recession: an updated systematic review and meta-analysis. BMC Oral Health.

[12] Garzón H., Suárez L.J., Muñoz S., Cardona J., Fontalvo M., Alfonso-Rodríguez C.A. (2022). Biomaterials used for periodontal disease treatment: focusing on immunomodulatory properties. Int J Biomater.

[13] Santonocito S., Ferlito S., Polizzi A. (2023). Impact exerted by scaffolds and biomaterials in periodontal bone and tissue regeneration engineering: new challenges and perspectives for disease treatment. Explor Med.

[14] Petrescu B.N., Mirica I.C., Miron R., Campian R.S., Lucaciu O. (2021). Platelet rich fibrin as a gingival tissue regeneration enhancer. J Dent Sci.

[15] Chaitra M.P., Shankar S.M., Shivakumar T.P., Gururaj S.B., Chidambar C.K., Bhushan K.S. (2024). Amniotic membrane versus platelet-rich fibrin in treatment of gingival recession- a randomized control trial. Saudi Dent J.

[16] Bhattacharya H.S., Gummaluri S.S., Rani A., Verma S., Bhattacharya P., R G.S.M. (2023). Additional benefits of titanium platelet-rich fibrin (T-PRF) with a coronally advanced flap (CAF) for recession coverage: a case series. Dent Med Probl.

[17] Lubaib M. (2023). A meta-analysis of amnion membrane in gingival recession. Bioinformation.

[18] Uzun B.C., Ercan E., Tunalı M. (2018). Effectiveness and predictability of titanium-prepared platelet-rich fibrin for the management of multiple gingival recessions. Clin Oral Invest.

[19] Nath J., Changmai A., Bhattacharjee K., Phukan A.H., Chakraborty D., Das U. (2022). Management of gingival recession by coronally advanced flap with and without amniotic membrane. J Pharm BioAllied Sci.

[20] Ozkal Eminoglu D., Arabaci T., Oztas Sahiner G.A. (2024). The effect of titanium-platelet rich fibrin on periodontal intrabony defects: a randomized controlled split-mouth clinical study. PLoS One.

[21] Law E.J., Taib H., Berahim Z. (2022). Amniotic membrane: an approach to periodontal regeneration. Cureus.

[22] Gummaluri S.S., Bhattacharya H.S., Astekar M., Cheruvu S. (2020). Evaluation of titanium-prepared platelet-rich fibrin and leucocyteplatelet-rich fibrin in the treatment of intra-bony defects: arandomized clinical trial. J Dent Res Dent Clin Dent Prospects.

[23] Sindgi R.P., Shah M.U. (2024). Comparative evaluation of coronally advanced flap with and without amniotic membrane in the treatment of localised miller's class I and class II gingival recession defects: a randomised controlled trial. J Clin Diagn Res.

[24] Vaswani B., Bajaj P. (2022). Titanium-platelet rich fibrin. J Res Med Dent Sci.

[25] Chakraborthy S., Sambashivaiah S., Kulal R., Bilchodmath S. (2015). Amnion and chorion allografts in combination with coronally advanced flap in the treatment of gingival recession: a clinical study. J Clin Diagn Res.

[26] Bolla V., Reddy P., Kalakonda B., Koppolu P., Manaswini E. (2019). Coronally advanced flap with amniotic membrane in the treatment of gingival recession: three case reports. Int J Appl Basic Med Res.

[27] Lafzi A., Abolfazli N., Faramarzi M., Eyvazi M., Eskandari A., Salehsaber F. (2016). Clinical comparison of coronally-advanced flap plus amniotic membrane or subepithelial connective tissue in the treatment of Miller's class I and II gingival recessions: a split-mouth study. J Dent Res Dent Clin Dent Prospects.

[28] Ustaoğlu G., Göller Bulut D., Gümüş K.Ç. (2020). Evaluation of different platelet-rich concentrates effects on early soft tissue healing and socket preservation after tooth extraction. J Stomatol Oral Maxillofac Surg.

[29] Gajul M., Bhate K., Awate S., Kakodkar P., Shah S. (2021). Comparative evaluation of the efficacy of wound healing with and without dehydrated human amniotic/chorionic membrane in alveoloplasty: a pilot study. J Korean Assoc Oral Maxillofac Surg.

